# COVID-19 and its impact on gynaecologic oncology practice in India—results of a nationwide survey

**DOI:** 10.3332/ecancer.2020.1067

**Published:** 2020-07-03

**Authors:** Anbukkani Subbian, Satinder Kaur, Viral Patel, Anupama Rajanbabu

**Affiliations:** 1 Comprehensive Cancer Center, Kovai Medical Center and Hospital, Coimbatore, 641014, India; 2 Department of Gynecologic Oncology, Dharamshila Narayana Superspecialty Hospital, Delhi, 110016, India; 3 Department of Gynecologic Oncology, Amrita Institute of Medical Sciences, Kochi, 682041, India; a https://orcid.org/0000-0003-2261-767X; b https://orcid.org/0000-0002-2885-8098

**Keywords:** COVID-19, gynaecological cancers, cancer surgery

## Abstract

The COVID-19 pandemic sweeping across the world has caused major disruptions in healthcare delivery and practice. A survey was conducted to assess the changes in the care of gynaecologic oncology patients in India.

**Methods:**

An online survey enquiring about the patient volumes and surgical load, and changes in practice protocols for endometrial, ovarian, cervical and vulval cancers was conducted in May, 2020.

**Results:**

The total number of responses received was 153. Barring duplicates, 148 were analysed. There was a significant drop in gynaecologic oncology patients attending government hospitals as compared to the non-government sector. The drop was not significantly different in areas having low versus high COVID-19 case volumes. The treatment of endometrial cancers remained the same although there was a marked shift from minimal access surgery to conventional surgery. Advanced ovarian cancer was mostly managed by neoadjuvant chemotherapy. Cervical and vulval cancer management remained the same, but radiotherapy protocols were modified by most.

**Conclusion:**

Based on clinician responses, it appears that most practices across India have suffered a fall in patient volumes. The responses from government sectors point towards a bigger hit in this segment of practice. While the management of endometrial cancers and cervical cancers was mostly unchanged, most cases of advanced ovarian cancer received neoadjuvant chemotherapy. Cervical cancer, when managed by chemoradiation, was likely to have altered radiation schedules.

## Background

The year 2020 is the year of COVID-19. The pandemic has affected 212 countries and territories around the globe and continues to ravage many. The US and many European and Asian countries felt the disease impact before India did. As per the Ministry of Health and Family welfare, the government of India statistics on 20th May 2020, 61,149 active cases of the novel Corona virus disease (COVID -19) caused by the SARS-COV-2 virus were reported in India. As many as 3,303 people had died of this disease in India [1]. The pandemic continues to spread in India and has resulted in widespread, government-led, lockdown measures to curb rapid spread. Health resources have been redirected towards care of COVID-19 patients in many centres here.

Drastic changes in healthcare delivery along with a country wide travel and movement ban means that many patients suffering from other health problems, notably cancer, are unable to access healthcare. Initial reports from China, the country which originally reported the virus, have evidence pointing towards potentially adverse outcomes in cancer patients affected by COVID-19 [2]. All these factors have led to a dramatic change in practice protocols for Gynaecologic cancer patients across the country. Organisations have issued guidelines, albeit based on limited evidence, that decisions regarding surgery or chemotherapy should be highly individualised. It has been suggested that clinicians should carefully consider the risks of COVID-19 infection during therapy and balance it with the risks of altering the prognosis of cancer due to delays in the treatment [3]. Based on these changes, this study was designed to look into the changes in gynaecologic oncology practice across India during the COVID-19 pandemic.

## Methods

This national survey was conducted amongst healthcare professionals involved in the care of gynaecologic cancer patients, when the caseload started rising steadily in several parts of the country. It was conducted in the first week of May, 2020. An online questionnaire was designed using google docs (https://docs.google.com). The questionnaire had six sections—Introduction, General questions, Endometrial cancer, Ovarian cancer, Cervical cancer and Vulval cancer in that order. The answers were given in multiple choice format and some were open ended and descriptive. The content was aimed at analysing the changes in the management of the above mentioned gynaecologic cancers types.

The survey was sent online by email and through social media platforms to clinicians with contact details accessed through national and local professional organisations. Attempts to make it representative of the entire country included telephone conversations with heads of leading cancer institutions across all states and ensuring participation. Survey responses were downloaded and analysed using SPSS software (v.20.0 IBM corporation, USA). To test categorical data, Chi square or Fisher’s Exact test was used. *p* value of <0.05 was considered to be significant.

## Results

The total number of responses recorded was 153. Five responders had double entries. The duplicates were omitted bringing down the responses for final analysis to 148. Responses were received from 19 out of 29 states in India.

Table 1 shows the participants in the survey and the general measures adopted during the pandemic. Most of the participants were from academic institutes (75.7%). Seventy seven percent of the participants were surgeons (including gynaecologic and surgical oncologists and gynaecologists doing gynaecologic cancer surgery) and the rest included medical and radiation oncologists who are involved in the care of gynaecologic cancer patients. Ninety six percent reported a decrease in clinical practice during this period with surgeries declining for 98% of the responders. Multidisciplinary tumour board meetings were discontinued in half the centres and when it was continued, 99% had made modification to switch to virtual platform or reduce the number of participants. PCR to detect active COVID-19 infection was done by most (84%) before start of the treatment. 93% of the surgeons used additional protective measures in the operating theatre but full personal protective equipment was used only by 4%. Forty-two percent of the surgeons used smoke evacuators during surgery.

Table 2 shows the differences in practice between the government and non-government practitioners. The comparison shows that during the pandemic there was significant reduction in the surgical volume of the practitioners in government institutions compared to those practicing in private institutions.

There was an equal number of responses from doctors practicing in areas with high and low volumes of COVID-19. A comparison between high and low COVID-19 incident states showed that the reduction of gynaecologic cancer patient load was seen across the country (Table 3). Healthcare facilities had a drop in cancer patient volumes irrespective of the COVID-19 case load in their states.

With respect to the change in protocols for each gynaecologic cancer type, the results are as follows:

### Endometrial cancer

Management of low risk, low-grade endometrial cancers was still predominantly surgical (86% offering hysterectomy). The rest were being managed by hormonal treatment. Most clinicians responded that delay of surgery could be done safely up to 6 weeks (73%). Regarding the management of advanced endometrial cancers, systemic chemotherapy was being offered by 46.9%. The surgery was being offered by about one third (36%) and 11.6% were offered upfront radiation therapy.

**Figure 1.**
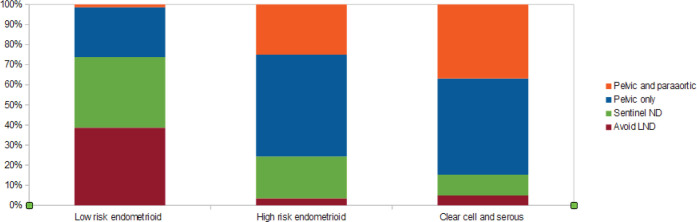
Lymph node dissection and type offered for endometrial cancer staging during current practice.

Lymphadenectomy forms an important part of endometrial cancer surgery. Figure 1 gives the proportion and type of lymph node assessment that was being offered during surgery.

### Ovarian cancer

Undiagnosed ovarian mass with high index of suspicion was likely to be managed by laparotomy by over a half of the clinicians. To avoid surgical risk, almost 30% suggested Neoadjuvant chemotherapy (NACT) following image guided biopsy of the mass. Regarding the safe interval for postponement of surgery in apparently early stage ovarian cancer, about 90% of the responses agreed that surgery should be offered within 6 weeks. Single or combination chemotherapy given for six cycles was still being preferred by almost 90% of the clinicians for early stage ovarian cancer.

For management of advanced stage ovarian cancer, neoadjuvant chemotherapy seemed to be the preferred option (93%). Seventy percent opted to operate after three cycles while the rest were giving up to four to six cycles of NACT. Almost 15% preferred to continue with maintenance chemotherapy or metronomic therapy or just close follow up after six cycles of chemotherapy to avoid surgery during the pandemic period.

Up to 95% of newly diagnosed ovarian cancer patients were likely to have their chemotherapy initiated within 6 weeks of diagnosis.

### Cervical cancer

For early operable cervical cancer, 66% of the respondents agreed on open radical hysterectomy, while less than 10% were likely to choose minimal access surgery. About 9% of the clinicians opted for concurrent chemoradiation for management of early stage cervical cancers. Two thirds of the respondents had altered their radiation schedules following the COVID-19 situation.

**Figure 2.**
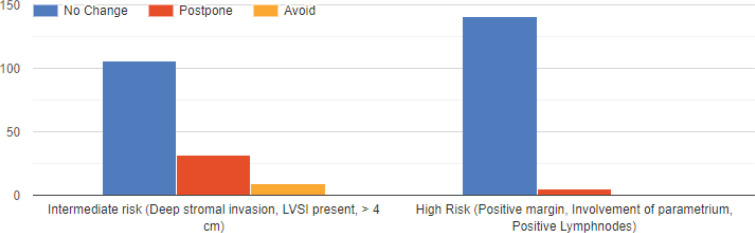
Present practice for adjuvant radiotherapy (VBT/EBRT) after radical hysterectomy for cervical cancer.

Figure 2 gives the responses obtained regarding post op adjuvant radiation therapy. The responses veered towards following pre-COVID-19 protocols of adjuvant radiation therapy for intermediate and high-risk factors by most.

### Vulvar cancer

For early stage vulvar cancer, a radical vulvectomy with sentinel node mapping (33%) was the most preferred option. While vulvectomy would be offered by the rest, 21% would not offer a groin node dissection and 19% would offer a concomitant node dissection.

For advanced vulvar cancer, concurrent chemoradiation was the preferred option by 42%. Neoadjuvant chemotherapy or radiation therapy before surgery was preferred by 43%. Surgery in this setting was the least preferred option.

## Discussion

The impetus behind this survey came from the sense of uncertainty that loomed large in conversations between clinicians across an array of platforms including on social media groups. It appeared that opinions were extremely diverse and this probably reflected the state of medical practice in India which ranges from small private, non-academic institutions to internationally known government or private oncology institutions, some of them with state-of-the-art facilities. The conversations were heavily coloured by the scientific data that was trickling in from countries which were at the peak of the COVID-19 pandemic ahead of India. The evidence from these countries pointed towards a potentially adverse outcome for COVID-19 infections in patients with cancer with a high risk of requiring ICU care, ventilation requirement and potentially death [2, 3]. Coupled with this, the inability of patients to access healthcare facilities due to the national lockdown meant that healthcare delivery is likely to be affected in many parts of the country. While health experts believe that the peak is yet to be reached in India, we needed to see the impact of the pandemic on gynaecologic cancer care at this time point [4].

It was apparent that healthcare delivery has been affected with more than 95% of the clinicians agreeing that the numbers of oncology outpatients and cancer surgeries had fallen to varying extents across the country. Few centres remained unaffected. For most patients, once diagnosed, it was highly likely that they would be offered the current standard of care treatment in all the cancer sites covered in this survey. The big change was seen in offering surgery on minimal access platforms for endometrial cancer. The reports of increased COVID-19 infection risk to the surgical team from aerosolised viral particles in pneumoperitoneum was probably a reason [5]. The current evidence on the relative COVID-19 risks to the surgical team for MIS and conventional surgery seems to be unclear [6]. However, considering the early recovery and reduced length of stay in MIS, it should be considered as a viable option. According to the SAGES guideline, consideration should be given to the possibility of viral contamination to staff during surgery whether open, laparoscopic or robotic and appropriate protective measures should be taken [7].

While surgery may be possible if there is no disruption of routine clinical services, delay in surgery may be inevitable in many centres because of diversion of healthcare resources towards COVID-19 cases. There is some evidence that a delay in surgery of more than 6 weeks was associated with the worst overall survival for type 1 endometrial cancers, especially stage 1 and 2 [8]. Keeping this in mind would be helpful when the time-lines for surgery are altered. Three fourths (75% approximately) of the respondents opined to operating within 6 weeks. Hormonal therapy was considered by 11% in this survey for treatment of early endometrial cancer. If a delay in surgery is inevitable it may be reasonable to offer systemic progesterone or intrauterine hormonal device [9]. For high risk endometrial cancer, most of the respondents favoured lymph node dissection, pelvic with or without paraaortic dissection. While this is in agreement with pre COVID guidelines, the increased surgical time and its associated increased morbidity in the present circumstances need to be considered. Current recommendations bring up the role of sentinel node dissection in these patients in line with the MSKCC protocol to reduce surgical morbidity [9, 10].

Early cervical cancer may be treated by surgery or chemoradiation as found appropriate. It had been shown that a delay of more than 4 months may significantly worsen prognosis across all stages of cervical cancers [11]. Hence, early and stage-appropriate treatment is more important than the modality of treatment, especially in this country with large cervical cancer volumes. While surgery seems more appropriate for early ovarian cancer, neoadjuvant chemotherapy that may be stretched to six cycles seems to be the recommendation for advanced ovarian cancers in the current pandemic situation [8, 10]. Most responses in this survey as described above seem to be in line with the current leading recommendations.

At present, it appears that the volume of cancer patients in centres across the country had reduced. This may lead to patients having delayed diagnosis and treatment and further lead to a possibility of presenting at more advanced and potentially incurable stages of disease in the near future. The hope is that easing of lockdown restrictions will enable patients to access healthcare facilities and enable timely treatment. The failure to do so will greatly increase the collateral damage of this unprecedented COVID-19 pandemic.

## Conclusions

This survey showed that there is a drop in the volume of cancer patients reaching treatment centres across India regardless of local COVID-19 volumes and the type of practice set up. While the management of endometrial cancers and cervical cancers was mostly unchanged, most cases of advanced ovarian cancer received neoadjuvant chemotherapy. Cervical cancer, when managed by chemoradiation, was likely to have altered radiation schedules.

## Conflicts of interest

The authors declare that there are no conflicts of interest.

## Funding statement

The article has not received any direct funding.

## Authors’ contributions

AS, SK, VP and AR were involved in designing the study. VP and AR did the data analysis. AS and AR were involved in the preparation of the manuscript.

## Figures and Tables

**Table 1.** Details of the participants and general measures adopted during the COVID 19 pandemic.

Question	Answer	*N* (%) (Total *n* = 148)	Not answered	
**General section**				
Practice setting	Academic institute: Government	36 (24.3)		
	Academic institute: Private	76 (51.4)		
	Non academic: Private	34 (23.0)		
	Non academic: government hospital	2 (1.4)		
Specialty	Gynaecologic Oncologist	74 (50.0)		
	Surgical Oncologist	26 (17.6)		
	Medical Oncologist	21 (14.2)		
	Radiation Oncologist	12 (8.1)		
	Gynaecologist	15(10.1)		
Gynaecological cancer patients seen per month (before the onset of COVID-19)	<50	84 (56.8)		
	51–100	30 (20.3)		
	101–250	14 (9.5)		
	>250	20 (13.5)		
Gynaecological cancer surgeries per month (before the onset of COVID-19)	<10	41 (27.7)		28 (18.9)
	11–20	38 (25.7)		
	21–30	18 (12.2)		
	>30	23 (15.5)		
States with high volume of confirmed COVID–19 cases	>3,500	75 (50.7)		
	≤3,500	73 (49.3)		
Decrease in practice after COVID 19 Pandemic	No	6 (4.1)		
	Yes	142 (95.9)		
Decreased practice in percentage	No surgery	3 (2.0)		1 (0.7)
	<10% of usual volume	39 (26.4)		
	About 1/4th of usual volume	58 (39.2)		
	About 1/2 of usual volume	30 (20.3)		
	About 3/4th of usual volume	14 (9.5)		
	No change	3 (2.0)		
MDTB in COVID 19 pandemic	Not conducted	72 (48.6)		
	Conducted	76 (51.4)		
Mode of MDTB if Yes	Virtual tumour board	54 (36.5)		60 (40.5)
	Tumour board with <5 participants	23 (15.5)		
	Tumour board with 6–10 participants	9 (6.1)		
	As before	2 (1.4)		
RT PCR before cancer treatment	Yes	60 (40.5)		
	Only selected high risk patients as per Institutional policy	64 (43.2)		
	COVID 19 testing not mandatory	24 (16.2)		
Additional precautions in view of COVID 19 pandemic	No	13 (8.8)		
	Yes	135 (91.2)		
Various additional precaution	No precaution	1 (0.7)		25 (16.9)
	N95 mask and visor	65 (44)		
	Full PPE	51 (34.4)		
	Full PPE and reduce personnel in OT	6 (4)		
Smoke evacuator during surgery	No	80 (54)		5 (3.4)
	Yes	63 (42.6)		

**Table 2.** Differences in practice between Government and non government practitioners.

*n* = 148		Government Hospital (*n* = 38) *n* (%)	Private Hospital (*n* = 110) *n* (%)	*p* value
**General section**				
Gynaecological cancer patients seen per month before COVID-19 pandemic	<50	14 (36.8)	70 (63.6)	**<0.0001**
	51–100	10 (26.3)	20 (18.2)	
	101–250	2 (5.3)	12 (10.9)	
	>250	12 (31.6)	8 (7.3)	
Gynaecological cancer surgeries per month before COVID-19 pandemic	<10	9 (24.3)	32 (38.6)	0.191
	11–20	11 (29.7)	27 (32.5)	
	21–30	6 (16.2)	12 (14.5)	
	>30	11 (29.7)	12 (14.5)	
No of responses according to COVID volume	High volume of COVID cases >3,500 cases during survey ; 5 states (Maharashtra, Gujarat, Tamil Nadu, Delhi, Rajasthan).	17 (44.7)	58 (52.7)	0.396
	Low volume of COVID cases ≤3,500 cases during survey	21 (55.3)	52 (47.3)	
Decrease in practice after COVID 19 Pandemic	No	1 (2.6)	5 (4.5)	0.606
	Yes	37 (97.4)	105 (95.5)	
Decreased practice in percentage	No surgery	3 (8.1)	0 (0)	**0.016**
	<10% of usual volume	13 (35.1)	26 (23.6)	
	About 1/4th of usual volume	13 (35.1)	45 (40.9)	
	About 1/2 of usual volume	7 (18.9)	23 (20.9)	
	About 3/4th of usual volume	1 (2.7)	13 (11.8)	
	No change	0 (0)	3 (2.7)	
MDTB in COVID 19 pandemic	No	27 (71.1)	45 (40.9)	**0.001**
	Yes	11 (28.9)	65 (59.1)	
Mode of MDTB if Yes	Virtual tumour board	6 (42.9)	48 (64.9)	**0.028**
	Tumour board with <5 participants	8 (57.1)	15 (20.3)	
	Tumour board with 6-10 participants	0 (0)	9 (12.2)	
	As before	0 (0)	2 (2.7)	

**Table 3.** Difference in practice according to the Volume of COVID-19.

*n*=148		Practice in states with confirmed COVID cases >3500 (*n*=75) *n*(%)	Practice in states with confirmed COVID cases ≤3500 (*n*=73) *n*(%)	*p* value
**General Section**				
Decreased practice in percentage	No surgery	1(1.4)	2(2.7)	0.068
	<10% of usual volume	24(32.4)	15(20.5)	
	About 1/4 of usual volume	33(44.6)	25(34.2)	
	About 1/2 of usual volume	12(16.2)	18(24.7)	
	About 3/4 of usual volume	4(5.4)	10(13.7)	
	No change	0(0)	3(4.1)	
Mode of MDTB	Virtual tumour board	36(76.6)	18(43.9)	0.004
	Tumour board with <5 participants	10(21.3)	13(31.7)	
	Tumour board with 6-10 participants	1(2.1)	8(19.5)	
	As before	0(0)	2(4.9)	
RT PCR before cancer treatment	Yes	36(48)	24(32.9)	0.165
	Only selected high-risk patients as per institutional policy	29(38.7)	35(47.9)	
	COVID 19 testing not mandatory	10(13.3)	14(19.2)	

## References

[1] https://www.mohfw.gov.in/.

[2] Liang W, Guan W, Chen R (2020). Cancer patients in SARS-CoV-2 infection: a nationwide analysis in China. Lancet Oncol.

[3] Wang H, Zhang L (2020). Risk of COVID-19 for patients with cancer. Lancet Oncol.

[4] https://resistancemap.cddep.org/COVID19_India.php.

[5] Choi SH, Kwon TG, Chung SK (2014). Surgical smoke may be a biohazard to surgeons performing laparoscopic surgery. Surg Endosc.

[6] Zheng MH, Boni L, Fingerhut A (2020). Minimally invasive surgery and the novel coronavirus outbreak: lessons learned from Italy. Ann Surg.

[7] https://www.sages.org/recommendations-surgical-response-covid-19/.

[8] Dessai S, Nachankar A, Kataria P (2020). Management of patients with gynecological cancers during the COVID19 pandemic.. Cancer Res Stat Treat.

[9] Ramirez PT, Chiva L, Eriksson AGZ (2020). COVID-19 global pandemic: options for management of gynecologic cancers. Int J Gynecol Cancer.

[10] Akladios C (2020). Recommendations for the surgical management of gynecological cancers during the COVID-19 pandemic - FRANCOGYN group for the CNGOF. J Gynecol Obstet Hum Reprod.

[11] Shen SC, Hung YC, Kung PT (2016). Factors involved in the delay of treatment initiation for cervical cancer patients: a nationwide population-based study. Medicine.

